# Electrospun Silica-Polyacrylonitrile Nanohybrids for Water Treatments

**DOI:** 10.3390/membranes13010072

**Published:** 2023-01-06

**Authors:** Beata Malczewska, Paweł Lochyński, Sylwia Charazińska, Andrzej Sikora, Ramin Farnood

**Affiliations:** 1 Institute of Environmental Engineering, Wrocław University of Environmental and Life Sciences, pl. Grunwaldzki 24, 50-365 Wroclaw, Poland; 2 Department of Nanometrology, Faculty of Electronics, Photonics and Microsystems, Wroclaw University of Science and Technology, 50-372 Wroclaw, Poland; 3 Department of Chemical Engineering & Applied Chemistry, Faculty of Applied Science & Engineering, University of Toronto, 200 College St, Toronto, ON M5S 3E5, Canada

**Keywords:** electrospinning, silica, nanofiber, PAN

## Abstract

In this work, the removal of NOM (natural organic matter) as represented by humic acid by means of electrospun nanofiber adsorptive membranes (ENAMs) is described. Polyacrylonitrile (PAN) was used for the preparation of ENAMs incorporating silica nanoparticles as adsorbents. The addition of silica to the polymer left visible changes on the structural morphology and fibers’ properties of the membrane. The membrane samples were characterized by pure water permeability, contact angle measurement, SEM, XPS, and XRD. This study assesses the preliminary performance of PAN-Si membranes for the removal of natural organic matter (NOM). The membrane rejected the humic acid, a surrogate of NOM, from 69.57% to 87.5%.

## 1. Introduction

One of the most critical problems facing humanity today is the increasing level of water contamination, accentuated by its increased consumption. In addition, new contaminants are emerging in aquatic environments where they have not been sufficiently removed by conventional treatment. Membrane processes have become commonplace in many industries in recent years. In terms of water and wastewater treatment technology, membranes are used for their high selectivity and efficiency as well as being highly resistant to mechanical, chemical, and thermal factors. The biggest limitation to the wider use of membranes is their tendency to fouling. Fouling results in the loss of capacity and requires raising the frequency of both hydraulic and chemical membrane cleaning. It is a complex physicochemical phenomenon caused mainly by organic matter dissolved in water (and, in particular, inorganic and organic colloids, as well as biological growth and its by-products). In the last decade, new materials and new manufacturing processes have been developed to improve the performance of membranes, as well as innovative methods proposed for their synthesis and modification [1].

An electrospinning technique was developed for the fabrication of fibers. This technique includes the utilization of electrostatic forces where fibers are made from a polymer solution. This is a highly popular method for producing many different fiber morphologies including very fine diameters, and various porosities and pore sizes (from nanometers to micrometers), along with great mechanical strength, thanks to inter-fiber connections [2,3,4,5,6]. Many studies emphasize the versatility of this method of fiber production, even though there have also been numerous attempts to improve the quality of fiber in order to expand their applications [7]. Many studies indicate the impact of the following parameters: Taylor-cone formation, polymer molecular weight and concentration, solution viscosity flow rate, electric intensity, work distance, and air humidity in the electrospinning process and fiber quality [2,3,4,5,6]. Several modifications have been made in order to improve the fiber quality. Among those, various post-treatment methods or crosslinking of polymers were implemented [6,8,9].

Surface modification can enhance electrospun nanofibers when nanoparticles are incorporated in membranes [10]. Recently, the fabrication of mixed matrix membranes with dispersive nanoparticles incorporated into the continuous polymer matrix has been attracting attention [11]. To improve the fiber quality, different nanoparticles are mixed with polymers and then electrospun to produce the scaffold without a functionalization process [12]. Some studies have investigated the addition of materials, such as SiO_2_, Al_2_O_3_, CuO, and TiO_2_ in electrospun fibers to improve their performance [6,13,14,15]. The selection of a polymer that becomes a membrane matrix is also important, as it affects membrane parameters such as surface tension, electrical conductivity, and viscosity [16].

Polyacrylonitrile (PAN) has been widely used for the production of membranes because it displays a very adequate mechanical strength and chemical stability [17]. The addition of salt to the polymer blend enhances the specific surface area of the membrane, and the greater the surface area, as shown, the stronger the interaction between membrane materials, foulants, and cleaning agents. [18]. The surface roughness of an electrospun fiber can be modified by introducing wrinkles, grooves, and pits on its surface [19].

Many research groups have been working on fabricating PAN-based composite nan-ofibers [20]. Expanding on that, electrospinning can be utilized to encapsulate all kinds of molecules, such as small-size chemical molecules [21,22]; drugs [23]; proteins and peptides [24]; nutrition and nanoparticles in monolithic core-shell, tri-layer core-shell, and other complicated nanostructures. However, limited efforts have been devoted to the addition of inorganic particles in a polymeric solution for creating hybrid nanofibers, as well as their influence on the working processes and potential applications.

There are still many pending issues relating to the relationship between membrane properties and their structure, composition, and topography. The aim of this study is to assess the use of electrospinning in order to fabricate novel PAN membranes loaded with silica nanoparticles, and to verify the impact of nanoparticles on membrane morphology. A unique approach is presented by comparing selected surface roughness parameters relating to the measurement of individual membrane fibers and parametrically describing the membrane morphology. It allows for the extension of the existing contemporary knowledge on the topic of membrane roughness. In addition, the possibility of using PAN and PAN-Si membranes in the removal of natural organic matter (NOM) is determined, which represents an interesting new potential application of this type of membrane relevant to environmental engineering.

## 2. Materials and Methods

### 2.1. Reagents

Polyacrylonitrile (PAN) with an average molecular weight of 150 kDa was used in this study. The solvent for the electrospinning of the polymer blend was a reagent grade dimethylformamide (DMF). Silica particles were purchased from Sigma Aldrich (Burlington, MA, USA). The particle size was 40–75 μm, pore size: 70 Å, and surface area: 480 m^2^/g.

### 2.2. Membrane Fabrication

A polymer solution of 12% (PAN) in dimethylformamide (DMF) was heated overnight at 52 °C in an oven. After 24 h, the bottle was shaken manually for 5 min and then stirred for 24 h at room temperature to achieve homogeneity.

In a separate bottle, a 12 wt.% suspension of silica in DMF was prepared. ENM (electrospun nanofiber membranes) was prepared by mixing PAN and silica in a 2:1 mass ratio, and thus fabricating the ENM in a single step. To prevent agglomeration of the particles, solutions were sonicated for 20 min before electrospinning.

The employed electrospinning apparatus was a KH-1-1 type electrostatic spinning machine manufactured by Ji’nan Liang Rui Technology Co. (Ji’nan, China). The polymer solution was loaded into a 20 mL syringe with an 18-gauge needle tip. The electrospinning process was executed with a flow rate of 90 mL/h. The applied voltage between the needle and the collector drum was 25 kV, and the distance between them was fixed at 20 cm. The electrospun nanofibers were collected by a metal drum collector covered with aluminum foil. Due to the limitations of the spinning machine, only one concentration of the addition could be spun. A similar membrane fabrication procedure was applied by Soberman et al. [19].

### 2.3. Characterization of ENAMs

The morphology of the electrospun nanofibers was examined by means of the field emission scanning electron microscope SEM/Xe-PFIB FEI Helios PFIB (Eindhoven, The Netherlands).

The FT-IR was obtained with the use of Thermo Scientific™ Nicolet™ iZ™10 FT-IR (Madison, WI, USA) at wavelengths from 4000 to 400 cm^−1^ with a resolution of 4 cm^−1^.

The X-ray photoelectron spectroscopy (XPS) was acquired using ThermoFisher Scientific K-Alpha (Waltham, MA, USA).

Membrane wetting was examined using a KRUSS Drop Shape Analyzer DSA100E (Hamburg, Germany). A drop of distilled water (V = 10 µm) was applied to each filter membrane and the wetting angle was determined by measuring the shape of the drop. Measurements were made 10 times for each membrane. The mean value of the wetting angle and that of the standard deviation (SD) were determined for each sample.

Atomic force microscopy (AFM) images were acquired using a Dimension 3100 atomic force microscope with a NanoScopeV controller (Bruker/formerly Veeco/, Santa Barbara, CA, USA). The topography was measured in ambient conditions with TappingMode™ (TM). The semicontact silicon probes, RTESPW (Bruker, Sunnyvale, CA), were used with the following parameters: spring constant in ranges 20–80 N/m, a resonance frequency of 264–342 kHz, and a nominal tip radius of curvature smaller than 10 nm. The areas of 2 µm × 2 µm with resolution 512 × 512 pixels were scanned in order to obtain enough data to observe interesting features. The images were analyzed and processed using the Gwyddion software [25]. In order to obtain the relevant data range for quantitative processing, a three-point leveling was followed by extracting the areas, approx. 510 nm × 510 nm, covering the plateau of a single fiber. The data extraction was performed for scans covering fibers with a larger diameter, in order to work with a certain area, thus making it possible to reform the statistical analysis. As the next step, a line-wise correction was performed in order to reduce fiber instability. Finally, the cylinder distortion was removed in order to reduce its impact on the determination of quantitative roughness.

### 2.4. Filtration Tests

In the filtration experiments, the ENM membrane was placed in the cartridge unit (in a dead-end filtration mode at constant flux), and the feed water was introduced. The feed water and permeate were tested after each filtration test. Rejection efficiencies (R %) were calculated using Equation (1):

R(%) = (1 − C_p_/C_f_) × 100, (1)

where C_f_ and C_p_ are feed and permeate, respectively.

Membrane performance was evaluated according to the rejection of NOM from the feed water. Because NOM mostly consists of humic (humic acid and fulvic acid) and some non-humic fractions (such as carbohydrates, amino acids, and proteins), for the filtration tests the humic acid was used as feed water. The number of organic compounds contained in the feed water was measured using the total organic carbon (TOC) reagent set. The membranes were pre-compacted for 30 min before the feed water was introduced.

## 3. Results and Discussion

### 3.1. Morphology and Surface Analysis

All filters fabricated in this study were composed of heterogeneous nanofibers, while samples with Si included large nanoparticle agglomerations in the fibers.

**Figure 1:**
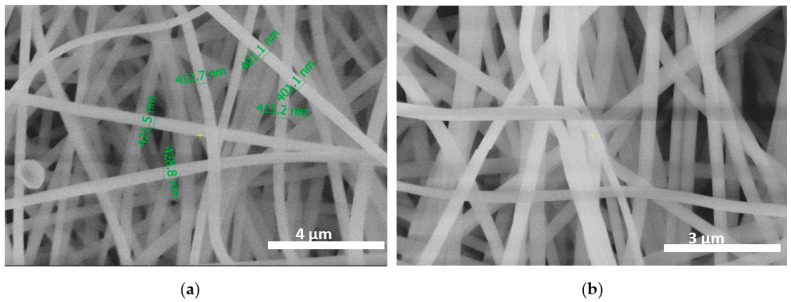
SEM images of (**a**) PAN linear nanofibers at ×4000; (**b**) PAN nanofibers at ×3000.

When the PAN concentration was above 12 wt.%, better spinnability was achieved and almost beadless fibers were formed (Figure 1a,b). The diameter of the fibers became more uniform and usually increased with the polymer concentration. The surface of the resultant fibers was relatively smooth. Regardless of the potential bending instability, the produced nanofiber mat had an almost uniform mat thickness (401 to almost 429 nm).

**Figure 2:**
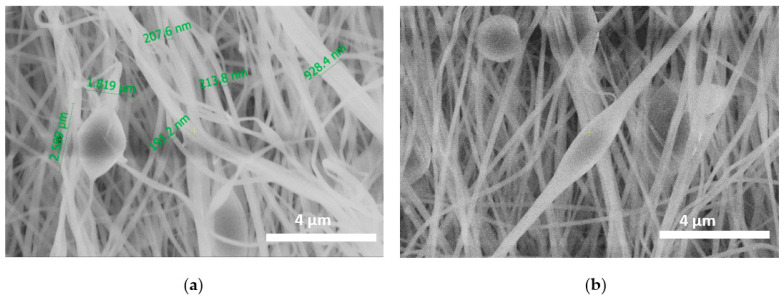
Si nanoparticles distribution in the PAN matrix: (**a**) SEM images of beads with nanoparticles; (**b**) beaded fibers.

When the PAN/DMF solution was mixed with silica particles, there was a significant difference in the fiber’s morphology. An adsorptive nanofiber membrane prepared via a one-step electrospinning of polyacrylonitrile blended with silica particles was shown to have large valleys and grooves on the surfaces of the membrane. The SEM image exhibited different degrees and modes of coverage for Si particles of different sizes when embedded in an ENM without size-fractionation (Figure 2a,b). The addition of Si particles changed the morphology of the membrane from smooth and straight nanofibers to branched nanofibers with elongated beads and broken fibers between beads. In the case of PAN-Si membranes, its fiber thickness reached several hundred nanometers up to 1.8 µm (Figure 2a).

The addition of nanoparticles into fibers is being widely studied so as to increase the applicability of electrospun membranes. Depending on the desired applicability, different particles have been added such as metal oxides, zeolites, enzymes, and adsorbents [23]. For example, Bortolassi et al. [9] evaluated the addition of silver to nanofibers of polyacrylonitrile (PAN) to be used as air filters. They reported that Ag nanoparticles gave the filters antibacterial properties. Hartati et al. also synthesized the electrospun membrane for air filtration by incorporating TiO_2_/Ag into the PAN matrix [26]. Khalili and Chenari successfully fabricated Zirconia-based ceramic nanofibers followed by calcination at different temperatures [27].

PAN adsorption membranes have been studied mostly for the adsorption of organic dyes from water. The other research direction is examining the ability of electrospun PAN membranes in heavy metal ion adsorption [6]. The addition of nanoparticles can provide nanofibers with improved properties such as hydrophilicity, toughness, and permeability [10]. Dong et al. [28] prepared electrospun SiO_2_/PVDF membranes and reported that the addition of the silica particles in the dope solutions contributed to a decrease in fiber diameter; however, at the same time, mechanical properties were enhanced [23,28].

PAN is a hydrophobic polymer and PAN electrospun membranes can be characterized by a high mechanical stability and water permeation flux [6]. According to Ebrahimi et al. the cylindrical structure and beadless surface of PAN membranes can be assigned to the application of the appropriate electrospinning conditions. In addition, they suggested that the distribution of fibers on the surface and between the PAN fibers acts as hydrophilic active sites, facilitating the absorption of water molecules through the fiber pores [29]. They, too, observed a near uniform distribution of fiber thickness for the PAN membrane. However, in contrast to the studies presented here, the PAN-Si membrane showed an increase in average fiber thickness. On the other hand, Jin et al. reported that the addition of SiO_2_ particles to PVA contributed to the creation of necklace-like structures [30].

Hou et al. [31] noticed that with the mass ratio of SiO_2_ increasing, membrane porosity slightly decreased, and pores shrunk, while membrane thickness was enhanced, resulting in an improvement to the salt rejection efficiency [9,30]. A similar observation was provided by Rasekh and Raisi [32], and this trend has been linked to the entrapment of nanoparticles in the fibers.

**Figure 3:**
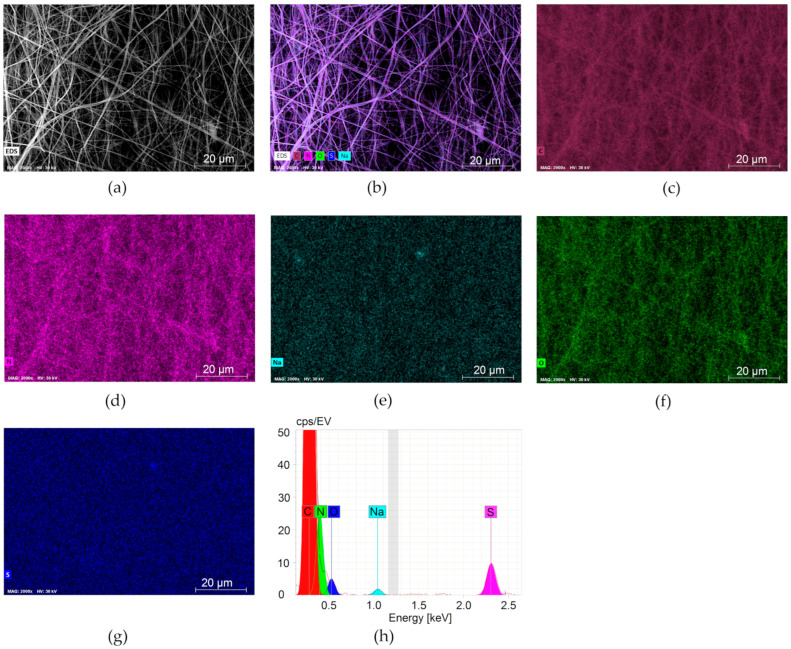
EDS elemental mapping images of PAN: (**a**) the SEM micrograph of the top membrane surface and the corresponding elemental mapping analyses of (**b**) mixed distribution of all elements; (**c**) carbon; (**d**) nitrogen; (**e**) sodium; (**f**) oxygen; (**g**) sulfur; (**h**) EDS spectrum of PAN membrane, containing elemental composition.

**Figure 4:**
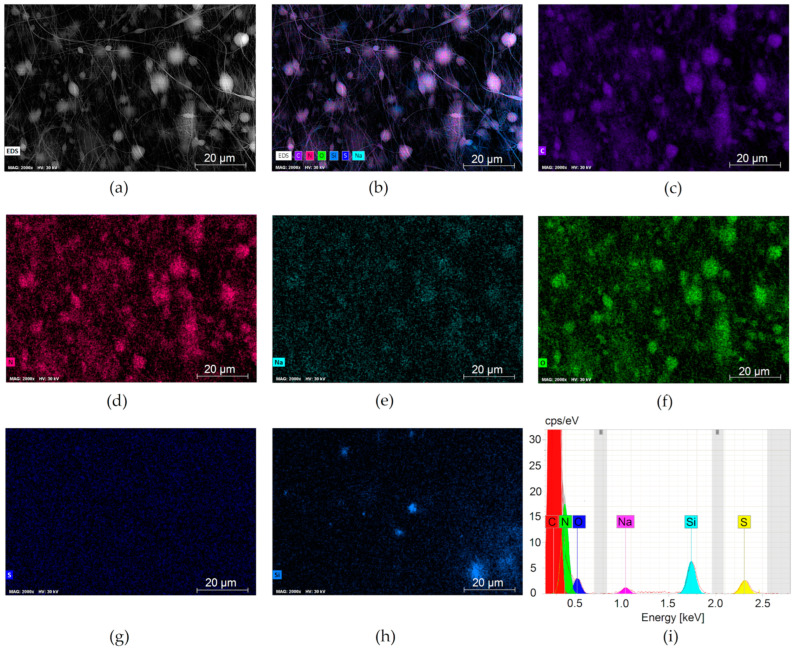
EDS of PAN-Si membrane: (**a**) The SEM micrograph of the top membrane surface and the corresponding elemental mapping analyses of (**b**) mixed distribution of all elements; (**c**) carbon; (**d**) nitrogen; (**e**) sodium; (**f**) oxygen; (**g**) sulfur; (**h**) silica; (**i**) EDS spectrum of PAN-Si membrane, containing elemental composition.

Figure 3 and Figure 4 depict the uniform distribution of SiO_2_ in the PAN-SiO_2_ membranes. The EDS elemental analysis of PAN and PAN-Si membranes are presented in Table 1 and Table 2. The addition of Si particles led to the appearance of new elements. This trend is inconsistent with the results reported by [23].

**Figure 5:**
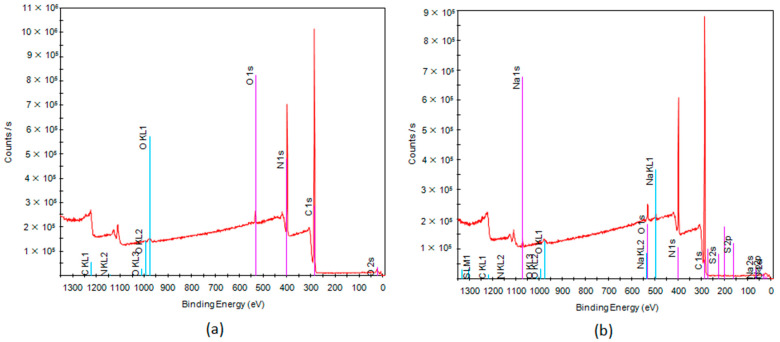
XPS survey spectra of (**a**) PAN, (**b**) PAN-Si membranes.

The XPS spectrum presented in Figure 5 depicts the atomic composition of the evaluated membrane. The relative concentration of the elements found in the pristine PAN membrane was as follows: carbon (71.5%), nitrogen (28.6%). Obviously, the PAN-Si membrane was characterized by a greater diversity of elements such as carbon (80.32%), nitrogen (15.39%), oxygen (4.07%), sodium (0.11%), and sulfur (0.11%). Silicon was not visible in the analysis above, as it can be assumed from the SEM images (Figure 4) that the vast majority of the Si particles were embedded within the fibers, forming characteristic fiber thickenings.

**Figure 6:**
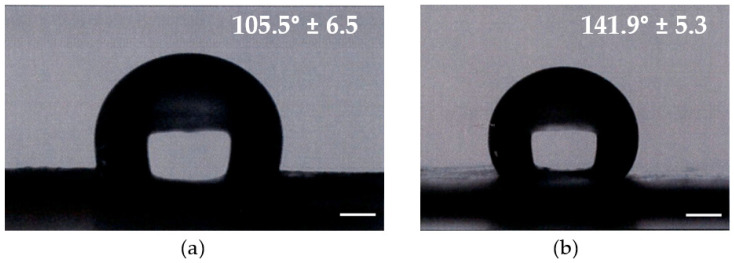
Contact angle of the tested membrane: (**a**) PAN-Si; (**b**) PAN.

The contact angle is used to quantify the wettability of a membrane surface, where a hydrophilic membrane is desired as it exhibits low fouling [32,33,34]. On a hydrophilic membrane, the wetting angle is less than 90 degrees, while on a hydrophobic membrane it exceeds 90 degrees. The addition of Si particles contributed to reducing the contact angle to 105.5° (SD ± 6.5) and 141.9° (SD ± 5.3) for the PAN-Si contact angle and PAN contact angle, respectively (Figure 6). We also performed a Student’s t-test to compare two analyzed membranes. The *p*-value was <0.00001 and the result was significant at *p* < 0.05. Therefore, it was statistically significant. The PAN-Si membrane had a lower average mean of the contact angle than PAN, which means PAN-Si showed better wettability when compared to the PAN membrane. The analysis of variance (ANOVA) was also used to compare both membranes at a 5% level of significance. The F-statistic value (129.7841) had a *p*-value of <0.00001, confirming that the results are statistically significant.

**Figure 7:**
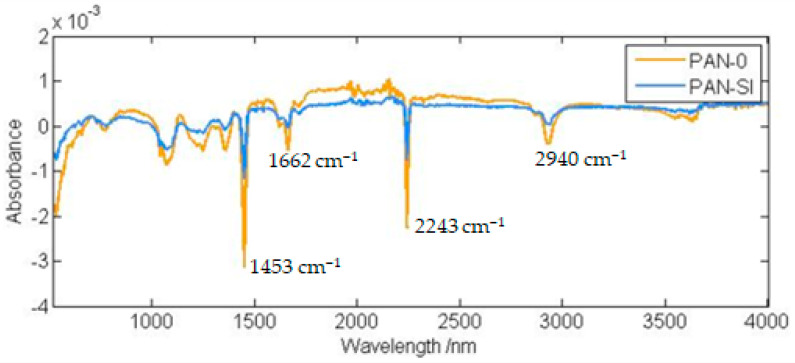
Normalized FTIR spectra of PAN and PAN-Si membranes.

When the FTIR results of PAN and PAN Si membranes were compared, reductions in the peaks were observed (Figure 7). The peak at 2940 cm^−1^ was assigned to –CH_2_-, while the bands of 2243 cm^−1^ and 1453 cm^−1^ indicated –C≡N groups [34]. The peak at 1662 cm^−1^ can be attributed to the C=O group in the amide structure [32,33,34,35,36].

The characteristic peaks for silica were observed at 798, 960, and 1100 cm^−1^ owing to Si-O-Si symmetric stretching, Si-OH stretching, and Si-O-Si asymmetric stretching vibrations, respectively.

Panda et al. noted that AFM showed that the membrane’s surface roughness was reduced with nanoparticle impregnation [35]. It has also been reported that the average surface roughness of the fibers impacts the adsorption efficiency [36].

**Figure 8:**
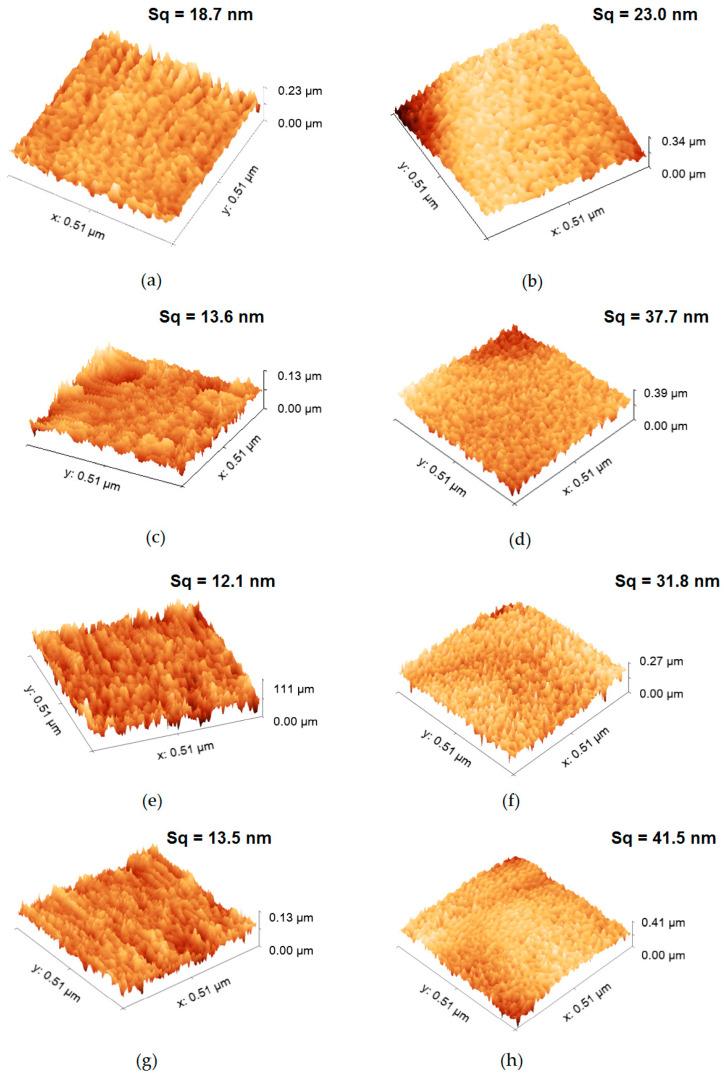
AFM images of single fiber fragments: (**a**,**c**,**e**,**g**) PAN membranes; (**b**,**d**,**f**,**h**) PAN-Si membranes.

Figure 8 displays the results of the AFM analysis, with special attention to single fibers so as to distinguish the surface of the membrane before and after modification. In order to provide a desired area that made it possible to perform the quantitative analysis, a fraction of scans of larger diameter fibers was chosen. The test area was appropriately selected to make sure that the tested roughness referred to the surface of a single fiber. Additionally, the data were processed in order to remove the cylinder shape-caused distortion. The surface morphologies and root mean square Sq represented the standard deviation of heights [37]. In order to reduce the impact of the materials’ non-homogeneity, the median values were calculated based on five different data sets. One can notice that the addition of silica particles has caused an increase in several surface roughness parameters (Figure 8). Individual measurements with a list of surface roughness parameters are summarized in the Supplemental Data (Table S1 in Supplementary Materials).

**Figure 9:**
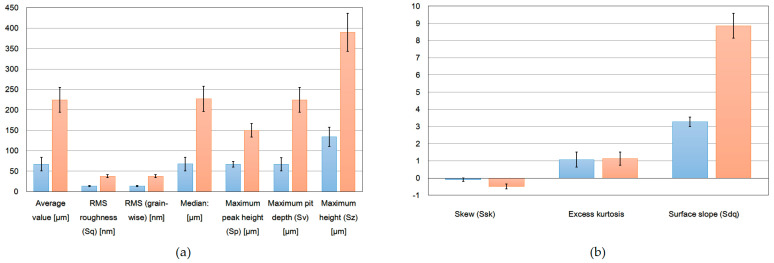
Surface roughness parameters of the fibers: (**a**) average value, RMS roughness, grain wise, median, maximum peak height, maximum pit depth, maximum height; (**b**) skew, excess kurtosis, surface slope. PAN−Blue bars, PAN−Si−Red bars.

Several areas in a series of images were acquired in order to provide statistically meaningful information. The most significant roughness parameters (Sa, Sq, Ssk, Sku, maximum peak height and pit depth, as well as the surface slope (Sdq)) were calculated (Figure 9). In order to reduce the influence of local morphology non-homogeneity, the median value of each parameter was determined. Additionally, the standard distribution was shown (as an error bar). The obtained values reveal a significantly larger roughness of the second set (silicon-implemented particles) of samples. The roughness parameter may be one of the most significant factors in terms of interaction with the environment. In particular, we consider the Sdr value as one that reveals a potentially active surface area, enabling a certain kind of interaction (chemical reactions, absorption, adhesion) [18,19,23,38,39,40,41,42]. To show the statistical significance between PAN and PAN-Si fibers, the Student’s t-test and ANOVA were used. The evaluation was made for the roughness equivalent (Sa). The t-value was 2.48098 and the *p*-value was 0.010615. The result was significant at *p* < 0.05. An analysis of variance (ANOVA) at a 5% level of significance was also used. The F-statistic value (6.15531) had a *p*-value of 0.02123, confirming that the results are statistically significant. The PAN-Si membrane had a lower average mean of Sa than PAN, which means PAN-Si had a higher surface roughness when compared to the PAN membrane.

**Figure 10:**
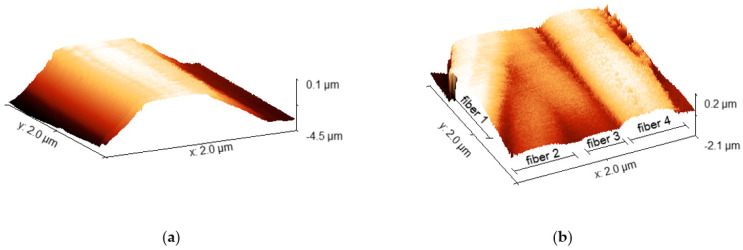
AFM images: (**a**) single PAN fiber and its surroundings; (**b**) four PAN-Si fibers and their surroundings.

The structure, composition, and fiber morphology of the electrospun membranes affected their properties in terms of the removal of natural organic matter (NOM). The study showed that the roughness parameters of the individual fiber fragments are more reliable and allow for a comparative analysis, in contrast to the AFM measurements found in the literature [18,19,32,37,38] for larger areas significantly exceeding the width of the fiber. Such wide-area measurements can only be considered for a preliminary assessment, although, due to the spaces involved, they give an unreliable indication of surface roughness parameters. It has to be underlined that large-scale AFM imaging may well be utilized in a general estimation of the fiber’s diameter, shape, and distance; however, such an analysis may be performed in a much more efficient manner using SEM imaging, as AFM may introduce too many distortions in the case of such complex structures (Figure 10). We also showed a 2 µm scan, revealing 4 parallel fibers and some space between them (Figure 10b). It has to be emphasized that the vertical distance between the following layers of fibers in the membrane may be impossible to determine using AFM, due to the limited penetration depth of the scanning tip.

In order to determine the parameters of a whole membrane structure and its interaction with the surroundings, one could first determine the fill factor (material volume vs. projected volume), which can be determined using weight, or more precisely, computer tomography. In addition, the way a single fiber is interacting with specific particles can be measured using the force spectroscopy technique, which is one of the diagnostic techniques related to scanning probe microscopy. Those methods will be employed in further investigations.

According to some researchers, the increase in surface roughness would provide a larger surface area which should lead to more feed water contact; however, due to the valley’s structure on the rougher surface, fouling tendencies are more significant [18,19,31].

### 3.2. Filtration Performances of PAN-Si

A major fraction of NOM is composed of humic substances that are responsible for the color of natural water [43]. Furthermore, a humic fraction has been identified as the major foulant in membrane water filtration [44].

To test the practical application capability of the PAN-Si membranes, a filtration of humic acid that represents NOM was conducted. The membrane PAN-Si showed a high rejection from 69.57% to 87.50% compared to the pure PAN membrane at only up to 15%.

The electrospun PAN membranes are characterized by a low-pressure build-up during filtration. In the filtration experiments, where HA was used as feed, two different fluxes (400 and 600 LMH) were applied to the PAN-Si membrane and no pressure increase was recorded. The top surface of the membrane turned from white to light brown as the filtration run proceeded. It suggests that much of the NOM rejection occurred at the very top of the membrane.

Filtration was conducted in a filtration unit with a dead end mode, such that carrying out multiple filtrations in this system was not possible, as each time the membrane needed to be removed from the unit, and a new method needed to be developed to be able to backwash it efficiently. Conducting filtration was associated with the cake formation, while a high constant flux filtration contributed to irreversible fouling. Chiu and Choo [45] suggested that the likely mechanism of NOM fouling is pore narrowing and blocking, followed by surface coverage and cake formation. Colloids having the same size deform via shear stress and applied pressure. Conventionally, the deposited layer on the membrane surface had a drawback due to serious fouling [46]. Therefore, a lot of research is being conducted to minimize this phenomenon and develop the most effective method of controlling it by a different pretreatment of the feed method, such as coagulation, oxidation, ion exchange, carbon adsorption, and mineral oxide adsorption [47].

## 4. Discussion

Liwen and Zhang also observed that with an increase in silica content in the PAN matrix, the dispersion of silica in the nanofiber changes from the homogeneous state to agglomeration that leads to the irregular surface morphology of nanofibers [20]. The addition of silica particles has an influence on the fiber diameter distribution. They also reported that the addition of silica nanoparticles also changes the thermal properties. This paper focuses solely on presenting the fiber characteristics of the membrane without checking its effectiveness in removing any impurities.

The filtration performance of the membrane is related to the structural factors of the nanofiber membrane such as fiber diameter, specific surface area, and fiber thickness. These parameters impact the filtration efficiency and pressure drop of the fiber membrane [48]. Fiber thickness is closely related to the filtration efficiency and pressure drop of the fiber membrane. Zhou et al. tested the applicability of the Kuwabara model to evaluate the filtration efficiency. According to their observation, the smaller the diameter, the greater the thickness, while a higher filtration efficiency for a single fiber is beneficial to the filtration efficiency of the fiber membrane [48]. Usually, the membrane thickness has a significant impact on the swelling degree and ion exchange capacity. Additionally, the ionic transfer is usually faster for thinner membranes. Reyes-Aguilera et al. reported an increase in the quantity of ion-exchange groups, and they associated this increase with the larger available surface area from the nanometric effect of the electrospun membrane [49].

Nanofibers have been reported to have a good specific surface area, porosity, and mechanical properties [6]. The mechanical characterization of the membranes helps determine the failure mechanism of the fibers during filtration. Different thermal and mechanical analysis techniques are used to investigate the membranes’ properties. Good mechanical properties often affect changes in membrane porosity. It has been reported that the porosity of composite membranes decreased as nanoparticle loading increased [50]. When the mechanical strength of membranes increased due to the addition of SiO_2_, that could be associated with a reduction in porosity [51]. Kim at al. tested the effect of the incorporation of SiO_2_ on the mechanical properties of electrospun nanofiber membranes. They discovered that the tensile strength and Young’s modulus were enhanced, which they attributed to SiO_2_ acting as a temporary crosslinking reagent between polymer chains [51]. On the other hand, Ullah et al. also reported good mechanical and thermal properties of PAN nanofibers, but the tensile strength decreased with the addition of silver sulfadiazine in PAN nanofibers during electrospinning [52]. When testing the strength of PAN fibers using a mechanotropic method, Varfolomeeva et al. reported high-strength PAN fibers, but the addition of silica led to a decrease in fiber strength [53]. The addition of inorganic particles into a polymer matrix can improve the mechanical property of the membrane. However, too much of such an addition in the matrix can lead to a decrease in tensile strength [54].

There are many approaches towards reducing the fouling, and one of them is the modification of the membrane surface with a focus on improving its selectivity, permeated flux, and antifouling properties in order to expand membrane applicability. Januário et al. proposed instead a new self-assembly method where particles were deposited layer-by-layer via electrostatic interaction through a pressure-assisted filtration system. They modified the membrane surface with sulfuric acid, titanium dioxide, and graphene oxide solutions [55]. The authors noted that in the modified membrane, a decrease in pore size was observed, although at the same time the antifouling characteristics of the membrane improved, and it had excellent separation properties for dyes [55]. While a layer-by-layer technique can be used to create multilayers with different components in each layer, it is also possible to incorporate multiple components into each individual layer [56]. Despite the undeniable advantages of this method of membrane modification, it has not been used in the filtration application area nor in any large-scale production.

## 5. Future Direction

Different techniques of spinning such as wet spinning, melt spinning, dry spinning, and electrospinning have been developed in order to prepare nanofibers in recent years. The wet spinning process offers the advantage of high production speed, while at the same time providing the biggest disadvantage of this approach, which is the difficulty of keeping the spinning condition constant. The major drawback of melt electrospinning is its slow throughput [57], even though it does not need any solvent. Syringeless electrospinning can be characterized by a higher productivity and processability, but the production efficiency of this method is quite low [58,59]. The jet spinning method can also be characterized by high production, but the fiber properties can be affected by the material’s characteristics and configuration of equipment [60]. Recently, a multi-needle technique has been developed to increase the range of applications in comparison to conventional electrospinning [6,61,62].

PAN electrospun nanofibers have a sizable specific surface area, high porosity, and good mechanical properties, as well as considerable application prospects [6]. However, the NOM fouling mechanism is not yet clear, and some potential modification of PAN mats remains to be developed. Further research is required to fabricate electrospun water purification membranes on an industrial scale with recyclability and reusability in the long term [63]. ENMs of a single polymer matrix have a limited application, as they are prone to pore wetting after long-term use due to low liquid entry pressure [64]. Therefore, various pre- and post-electrospinning modification strategies have been used for ENMs to increase the surface hydrophobicity and reduce pore size while maintaining higher permeate fluxes [65]. Another direction of research is the application of nanoparticles. The nanoparticles can be introduced in several ways, e.g., by blending with the electrospinning dope solutions, adhesive deposition or dip-coating, and electro-spraying of nanoparticles during the process of electrospinning [66]. A second one is the modification of the surface membrane by the application of a layer-by-layer self-assembly via electrostatic attraction. This approach has been widely investigated in biomedical applications.

Further investigations into the optimal design and operation of such process combinations that take into account source water qualities, membrane properties, adsorbent regeneration, and system operation flexibilities are needed.

## 6. Conclusions

Pure PAN nanofibers with a diameter range of 401 nm to 429 nm have been successfully synthesized through the electrospinning method. The morphology of the PAN nanofiber membrane shows beadless fibers at the uniform diameter of the fiber’s distribution. The addition of silica particles impacts the membrane morphology, while the PAN-Si nanofiber membrane is non-uniform with branched nanofibers and elongated beads.

The EDS and FTIR results have revealed that considerable Si parts of silica nanoparticles were incorporated into the membrane matrix.

The contact angle results have revealed that PAN-Si membranes have slightly reduced.

AFM measurements provided insights into the morphology of nanofibers. The analysis of several roughness parameters acquired from a set of measurement results revealed that Si particles caused an increase in surface roughness.

Adding Si particles resulted in the improvement of membrane performance due to changes in the morphology of fibers. The pure PAN membrane did not remove NOM satisfactorily but adding Si particles had an impact on membrane morphology as well as increasing NOM removal.

## Figures and Tables

**Table 1:** EDS elemental analysis of PAN membranes.

Element	At. No.	Netto	Mass [%]	Mass Norm [%]	Atom [%]	Abs. Error [%]	Rel. Error [%]
Carbon	6	2,969,112	48.42	48.42	52.85	4.92	10.17
Nitrogen	7	256,536	43.57	43.57	40.78	4.55	10.45
Oxygen	8	52,436	7.47	7.47	6.12	0.84	11.21
Sodium	11	20,324	0.19	0.19	0.11	0.04	19.67
Sulfur	16	127,571	0.34	0.34	0.14	0.04	10.98

**Table 2:** EDS elemental analysis of PAN-Si membranes.

Element	At. No.	Netto	Mass [%]	Mass Norm [%]	Atom [%]	Abs. Error [%]	Rel. Error [%]
Carbon	6	1,949,936	54	54	58.50	5.50	10.19
Nitrogen	7	176,585	39.63	39.63	36.81	4.18	10.54
Oxygen	8	31,439	4.98	4.98	4.05	0.58	11.63
Silicon	14	76,725	0.75	0.75	0.35	0.06	7.55
Sulfur	16	33,412	0.39	0.39	0.16	0.04	10.00
Sodium	11	13,328	0.25	0.25	0.14	0.04	16.44

## Data Availability

The data presented in this study are available on request from the corresponding author.

## Supplementary Materials

The following supporting information can be downloaded at: https://www.mdpi.com/article/10.3390/membranes13010072/s1, Table S1. AFM individual measurements of single fiber fragments: (a,c,e,g,i) PAN membranes; (b,d,f,h,j) PAN-Si membranes.
