# Supplementary material for: Electrospun Silica-Polyacrylonitrile Nanohybrids for Water Treatments
Source: Membranes (Basel). 2023 Jan 6;13(1):72. doi: 10.3390/membranes13010072 (PMC9861717; doi:10.3390/membranes13010072)
Supplement: Supplementary file 1 [file membranes-13-00072-s001.zip › membranes-2101219-supplementary.pdf]

membranes

MDPI

Supplementary Materials

Electrospun Silica-Polyacrylonitrile Nanohybrids for Water Treatments

Beata Malczewska [1,*], Paweł Lochyński [1], Sylwia Charazińska [1], Andrzej Sikora [2] and Ramin Farnood [3]

[1] Institute of Environmental Engineering, Wrocław University of Environmental and Life Sciences, pl. Grunwaldzki 24, 50-365 Wroclaw, Poland; pawel.lochynski@upwr.edu.pl (P.L.); sylwia.charazinska@upwr.edu.pl (S.C.)
[2] Department of Nanometrology, Faculty of Electronics, Photonics and Microsystems, Wroclaw University of Science and Technology, 50-372 Wroclaw, Poland; andrzej.sikora@pwr.edu.pl
[3] Department of Chemical Engineering & Applied Chemistry, Faculty of Applied Science & Engineering, University of Toronto, 200 College St, Toronto, ON M5S 3E5, Canada; ramin.farnood@utoronto.ca
* Correspondence: beata.malczewska@upwr.edu.pl

Table S1. AFM individual measurements of single fiber fragments: (a), (c), (e), (g), and (i) PAN membranes; (b), (d), (f), (h), and (j) PAN-Si membranes.

(a) PAN membrane

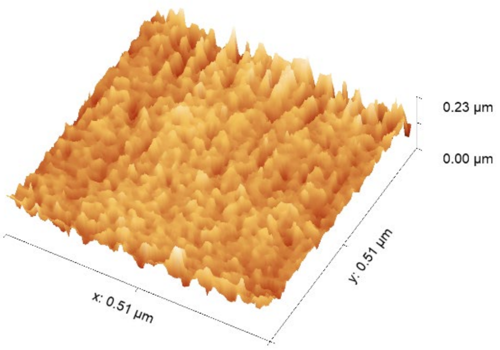

(b) PAN-Si membrane

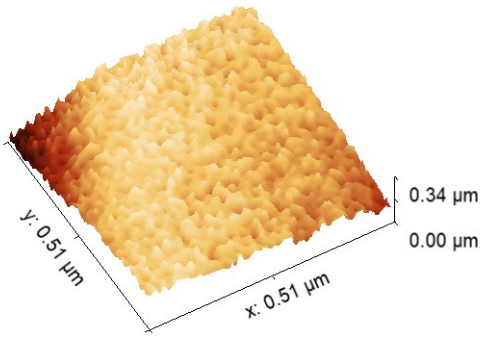

(a) PAN membrane		(b) PAN-Si membrane	
Statistical Quantities		Statistical Quantities	
Average value:	134.549 nm	Average value:	115.803 nm
RMS roughness (Sq):	18.7042 nm	RMS roughness (Sq):	23.0488 nm
RMS (grain-wise):	18.7042 nm	RMS (grain-wise):	23.0488 nm
Mean roughness (Sa):	13.9075 nm	Mean roughness (Sa):	18.1009 nm
Skew (Ssk):	−0.346433	Skew (Ssk):	−0.462895
Excess kurtosis:	2.55043	Excess kurtosis:	0.583787
Projected area:	257,874 nm^2	Projected area:	0.25787 µm^2
Surface area:	853,566 nm^2	Surface area:	1.10023 µm^2
Surface slope (Sdq):	4.44792	Surface slope (Sdq):	5.80853
Volume:	34.6967 × 10^{-21} m^3	Volume:	29.8625 × 10^{-21} m^3

**(c) PAN membrane**		**(d) PAN-Si membrane**	
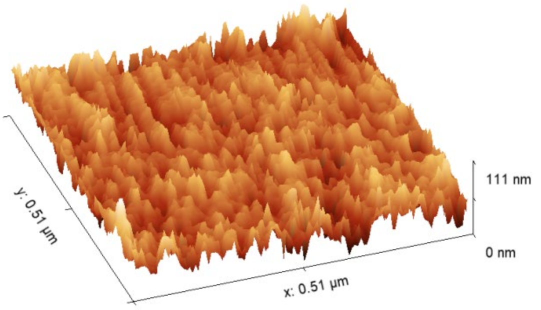		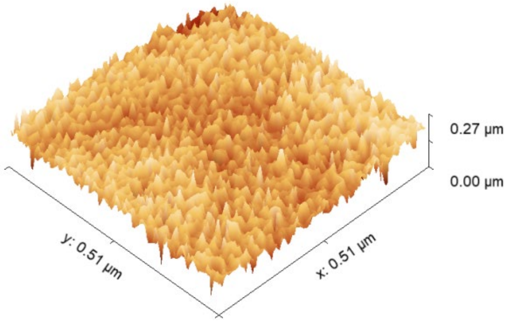	
Statistical Quantities		Statistical Quantities	
Average value:	49.814 nm	Average value:	161.991 nm
RMS roughness (Sq):	12.0975 nm	RMS roughness (Sq):	31.7688 nm
RMS (grain-wise):	12.0975 nm	RMS (grain-wise):	31.7688 nm
Mean roughness (Sa):	9.4858 nm	Mean roughness (Sa):	24.1850 nm
Skew (Ssk):	7.50480×10^{-3}	Skew (Ssk):	−0.486780
Excess kurtosis:	0.483549	Excess kurtosis:	1.20452
Projected area:	261,856 nm^2	Projected area:	0.26186 μm^2
Surface area:	712,632 nm^2	Surface area:	1.78483 μm^2
Surface slope (Sdq):	3.27822	Surface slope (Sdq):	9.00439
Volume:	13.0440×10^{-21} m^3	Volume:	42.4183×10^{-21} m^3

**(e) PAN membrane**	**(f) PAN-Si membrane**
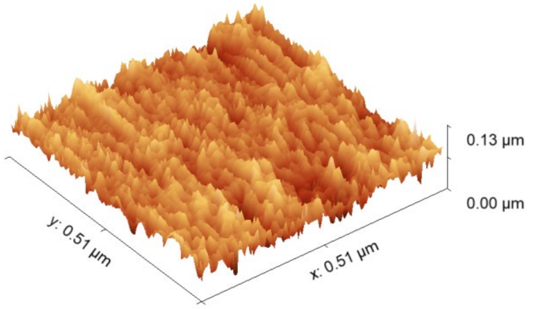	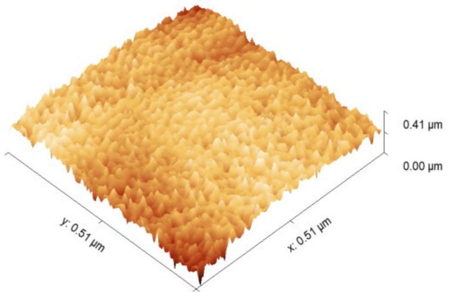
Statistical Quantities	Statistical Quantities
Average value: 64.863 nm	Average value: 262.491 nm
RMS roughness (Sq): 13.4755 nm	RMS roughness (Sq): 41.4914 nm
RMS (grain-wise): 13.4755 nm	RMS (grain-wise): 41.4914 nm
Mean roughness (Sa): 10.5473 nm	Mean roughness (Sa): 32.0846 nm
Skew (Ssk): −0.179237	Skew (Ssk): −0.503540
Excess kurtosis: 0.296227	Excess kurtosis: 0.886592
Projected area: 261,856 nm^2	Projected area: 0.26186 μm^2
Surface area: 774,965 nm^2	Surface area: 1.88802 μm^2
Surface slope (Sdq): 3.60174	Surface slope (Sdq): 9.42221
Volume: 16.9848×10^{-21} m^3	Volume: 68.7349×10^{-21} m^3

**(g) PAN membrane**		**(h) PAN-Si membrane**	
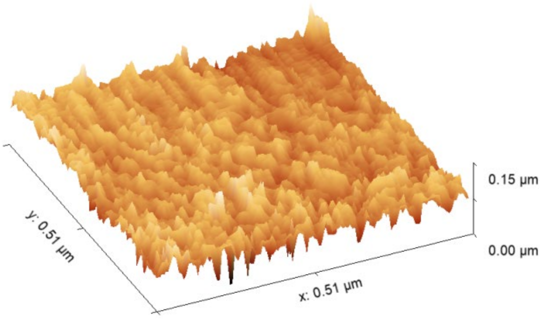		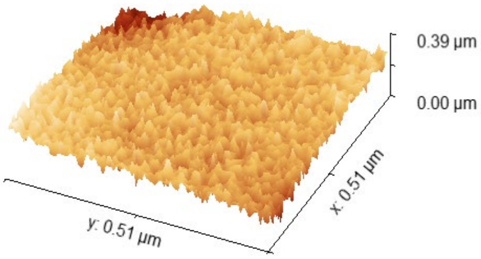	
Statistical Quantities		Statistical Quantities	
Average value:	82.114 nm	Average value:	239.720 nm
RMS roughness (Sq):	11.5133 nm	RMS roughness (Sq):	38.8378 nm
RMS (grain-wise):	11.5133 nm	RMS (grain-wise):	38.8378 nm
Mean roughness (Sa):	8.9604 nm	Mean roughness (Sa):	28.1871 nm
Skew (Ssk):	0.0910920	Skew (Ssk):	−1.01578
Excess kurtosis:	1.07171	Excess kurtosis:	2.61707
Projected area:	261,856 nm^2	Projected area:	0.26186 µm^2
Surface area:	682,607 nm^2	Surface area:	1.76057 µm^2
Surface slope (Sdq):	3.16464	Surface slope (Sdq):	8.85958
Volume:	21.5021 × 10^{-21} m^3	Volume:	62.7722 × 10^{-21} m^3

(i) PAN membrane **(j) PAN-Si membrane**

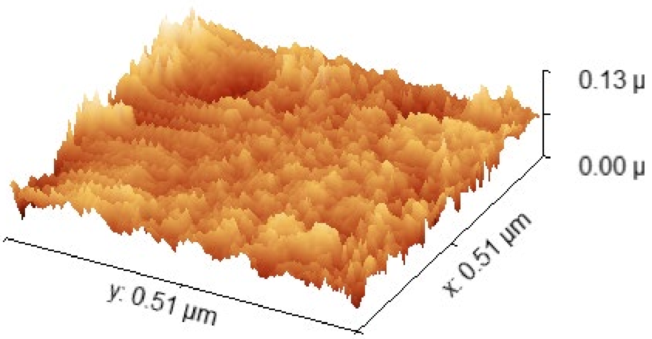

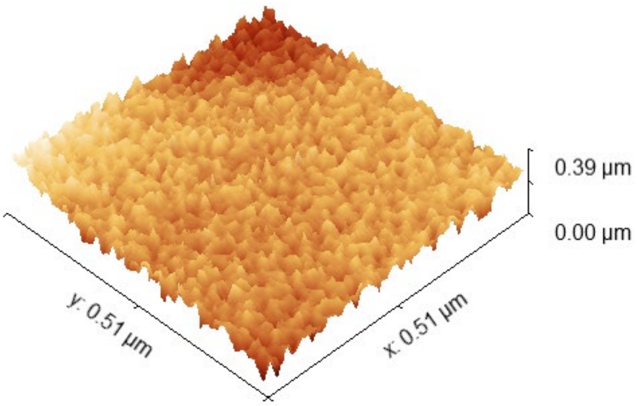

Statistical Quantities		Statistical Quantities	
Average value:	67.237 nm	Average value:	224.321 nm
RMS roughness (Sq):	13.5971 nm	RMS roughness (Sq):	37.6789 nm
RMS (grain-wise):	13.5971 nm	RMS (grain-wise):	37.6789 nm
Mean roughness (Sa):	10.3022 nm	Mean roughness (Sa):	28.3934 nm
Skew (Ssk):	-25.8007×10^{-3}	Skew (Ssk):	−0.278625
Excess kurtosis:	1.07242	Excess kurtosis:	1.13166
Projected area:	261,856 nm^2	Projected area:	0.26186 μm^2
Surface area:	701,993 nm^2	Surface area:	1.73121 μm^2
Surface slope (Sdq):	3.22295	Surface slope (Sdq):	8.64715
Volume:	17.6063×10^{-21} m^3	Volume:	58.7399×10^{-21} m^3
